# A genetic variant controls interferon-β gene expression in human myeloid cells by preventing C/EBP-β binding on a conserved enhancer

**DOI:** 10.1371/journal.pgen.1009090

**Published:** 2020-11-04

**Authors:** Anaïs Assouvie, Maxime Rotival, Juliette Hamroune, Didier Busso, Paul-Henri Romeo, Lluis Quintana-Murci, Germain Rousselet

**Affiliations:** 1 Laboratoire Réparation et Transcription dans les cellules Souches, UMRE008 Stabilité Génétique Cellules Souches et Radiations, Université de Paris, Université Paris-Saclay, CEA/IRCM, Inserm U1274, Fontenay-aux-Roses, France; 2 Unit of Human Evolutionary Genetics, CNRS UMR2000, Institut Pasteur, Paris, France; 3 Plate-forme Génomique, Université de Paris, Institut Cochin, CNRS, INSERM, Paris, France; 4 CIGEx, UMRE008 Stabilité Génétique Cellules Souches et Radiations, Université de Paris, Université Paris-Saclay, CEA/IRCM, Inserm U1274, Fontenay-aux-Roses, France; 5 Chair Human Genomics & Evolution, Collège de France, Paris, France; The Jackson Laboratory, UNITED STATES

## Abstract

Interferon β (IFN-β) is a cytokine that induces a global antiviral proteome, and regulates the adaptive immune response to infections and tumors. Its effects strongly depend on its level and timing of expression. Therefore, the transcription of its coding gene *IFNB1* is strictly controlled. We have previously shown that in mice, the TRIM33 protein restrains *Ifnb1* transcription in activated myeloid cells through an upstream inhibitory sequence called ICE. Here, we show that the deregulation of *Ifnb1* expression observed in murine *Trim33*^-/-^ macrophages correlates with abnormal looping of both ICE and the *Ifnb1* gene to a 100 kb downstream region overlapping the *Ptplad2*/*Hacd4* gene. This region is a predicted myeloid super-enhancer in which we could characterize 3 myeloid-specific active enhancers, one of which (E5) increases the response of the *Ifnb1* promoter to activation. In humans, the orthologous region contains several single nucleotide polymorphisms (SNPs) known to be associated with decreased expression of *IFNB1* in activated monocytes, and loops to the *IFNB1* gene. The strongest association is found for the rs12553564 SNP, located in the E5 orthologous region. The minor allele of rs12553564 disrupts a conserved C/EBP-β binding motif, prevents binding of C/EBP-β, and abolishes the activation-induced enhancer activity of E5. Altogether, these results establish a link between a genetic variant preventing binding of a transcription factor and a higher order phenotype, and suggest that the frequent minor allele (around 30% worldwide) might be associated with phenotypes regulated by IFN-β expression in myeloid cells.

## Author summary

Genome-wide association studies identify multiple genetic variants associated with higher order phenotypes. Pinpointing the causative variant and understanding its molecular mode of action is a complex task. Using a murine model of interferon-β transcriptional deregulation, we characterize a super-enhancer controlling *Ifnb1* expression in myeloid cells. The most active enhancer of this locus is conserved in humans, but presents a frequent variant found in around 30% of the population worldwide. This variant prevents binding of the C/EBP-β transcription factor, and is associated with decreased expression of *IFNB1* in activated monocytes. When mimicked in the murine enhancer, it abolishes its inducible enhancer activity. Our results describe the molecular link between a point mutation and a cellular phenotype that could influence clinical situations.

## Introduction

The induction of type I interferons, and in particular of interferon beta (IFN-β), is an essential step of the antiviral response [1]. The transcription of the IFN-β coding gene, *IFNB1*, is rapidly induced upon viral infection through multiple pathways sensing virus-derived nucleic acids [2]. The released IFN-β protein is able to induce the expression of antiviral proteins encoded by Interferon Stimulated Genes (ISGs) [3,4], that interfere with the infection of the cell by other viruses, hence the name interferon. In addition, IFN-β targets immune cells, facilitating the induction of an efficient adaptive immune response [2]. It promotes the activation and the T cell stimulatory capacity of dendritic cells [5,6], and has direct co-stimulatory properties on T cells, in particular by stimulating their proliferation once they have been activated by engagement of the T cell receptor and of co-stimulatory receptors [7]. Interestingly, it has now been recognized that radiotherapy favors an anti-tumor immune response through a pathway involving IFN-β [8–12].

The timing of interferon expression is of primary importance for the immune response. For instance, the relative timing of IFN-β stimulation and T cell receptor (TCR) activation decides the targeted T cell response [13]. If the IFN-β signal precedes the TCR activation, it will lead to STAT1 activation and apoptosis, whereas a previous TCR activation will decrease STAT1, leading to IFN-β signaling through Stat4/Stat5 and T cell proliferation. In addition, whereas an acute IFN-β signal is pro-inflammatory, a prolonged signal, such as the one observed during chronic infection, leads to immune suppression. Dysregulated type I interferon expression is the molecular substrate of Aicardi-Goutières syndrome [14], an early onset brain disease associated with signs of inflammation. Therefore, the molecular control of *IFNB1* transcription has been the subject of intense research.

The pioneering work performed in the laboratories of T. Maniatis and D. Thanos allowed to identify the enhanceosome, a promoter proximal structure that serves as a docking site for the cooperative binding of several transcription factors involved in *IFNB1* transcriptional control, including NF-κB (p50:relA), ATF2:c-jun, and IRF3/IRF7 [15–17]. Several regions upstream or downstream from the *IFNB1* gene were described to positively or negatively regulate *IFNB1* transcription by fixing factors such as NF-κB [18], XBP-1 [19], YY1/2 [20,21], or β-catenin [22]. In particular, Banerjee et al. identified a region located 20 kb upstream from the human *IFNB1* gene that loops to the *IFNB1* promoter, binds phospho-IRF3, and is required for *IFNB1* induction upon virus infection in fibroblasts [23]. We have shown that in murine macrophages, this locus binds the TRIM33 protein, and inhibits *Ifnb1* transcription at the end of activation by lipopolysaccharide (LPS) or poly(I:C), targeting Toll-like receptors 4 and 3, respectively [24]. We have therefore called it ICE for *Ifnb1* Control Element. Knocking out *Trim33* in macrophages leads to an increased binding of the acetyl-transferase CBP/p300 on the *Ifnb1* promoter, an increased acetylation of histone H3, and an over-expression of *Ifnb1* at late stages of activation [24].

TRIM33 belongs to the TRIM family of proteins, that harbor at their N terminus a TRIpartite Motif composed of a Ring domain followed by 2 B boxes and a coiled-coil domain, and that generally carry an ubiquitin ligase function [25]. TRIM33, like TRIM24, TRIM28 and TRIM66, also contains a PHD domain and a Bromo domain at its C terminus that allow binding to modified histones [26]. This chromatin reader property, associated with its capacity to interact with various transcription factors, gives TRIM33 an important function in hematopoiesis [27–30]. In particular, TRIM33 interferes with the formation of the SMAD complexes in the TGF-β pathway, although its precise function, and whether it relies on SMAD4 ubiquitination, is still debated [31–34]. Although we have shown that the ubiquitin ligase activity of TRIM33 is not required to control *Ifnb1* expression in myeloid cells [24], the precise mechanism of this regulation is not known.

In this manuscript, we show that the absence of TRIM33 in activated murine myeloid cells correlates with an increased looping of the *Ifnb1* promoter and ICE to a region overlapping the 100 kb downstream gene *Ptplad2*/*Hacd4*. We characterize this region as a myeloid super-enhancer. In addition, we identify the human orthologous region as a locus harboring several single nucleotide polymorphisms (SNPs) previously associated with quantitative differences in *IFNB1* expression in human monocytes activated with lipopolysaccharide (LPS). The minor allele of the most associated SNP disrupts a conserved C/EBP-β binding site and prevents C/EBP-β binding. In a human context, or when mimicked in the murine sequence, it blocks the inducible enhancer activity of a DNA fragment encompassing the C/EBP-β binding site. Our results identify a new conserved *IFNB1* enhancer, and functionally link its polymorphism to a cellular phenotype.

## Results

### Abnormal looping of the *Ifnb1* locus in activated *Trim33*^-/-^ macrophages identifies a myeloid super-enhancer within the *Ptplad2* gene

Two reports have used chromatin immunoprecipitation followed by deep sequencing (ChIP-seq) to identify chromatin peaks bound by both TRIM33 and the CCCTC binding factor (CTCF), a DNA looping organizing protein, in embryoid bodies [35] and male germ cells [36]. Because CTCF and cohesin, another DNA looping organizing protein complex, regulate gene expression, we hypothesized that they could participate in the control of *Ifnb1* expression by TRIM33. We first tested whether TRIM33, CTCF and cohesin co-localized on the chromatin of myeloid cells. We performed ChIP-seq targeting CTCF and RAD21, a subunit of cohesin, in the murine macrophage cell line RAW264.7 cells, and aligned the observed peaks with TRIM33 peaks [24]. Nearly 15% of CTCF peaks were also bound by RAD21 and TRIM33 (cluster 2 in S1A Fig), including 2 peaks located just upstream from the ICE *Ifnb1* regulatory region (S1B Fig). We went on by studying a potential role of CTCF in the interferon response of myeloid cells, using transcriptome data comparing activated *Ctcf*^-/-^ and wild-type (WT) bone marrow derived macrophages (BMDMs) [37]. Gene ontology (GO) cluster analysis of the 200 most differentially expressed genes revealed an important remodeling of the inflammatory response, with inflammatory response genes being significantly enriched in both under- and over-expressed genes (S1C Fig). However, ISGs, including Rsad2, Mx1, Mx2, Ifitm6, Ifi205, Isg20, or Cxcl11, were present only in under-expressed genes. The most enriched GO cluster identified from genes under-expressed in *Ctcf*^-/-^ macrophages included ‘cellular response to interferon beta’ (p < 5x10^-5^; S1C Fig), and was not enriched in over-expressed genes. A second GO term cluster including ‘response to virus’ was also specifically enriched in under-expressed genes (p < 5x10^-5^) (S1C Fig). These analyses demonstrate that in myeloid cells, CTCF is required for a full-blown interferon response, and is partially co-localized with TRIM33.

**Fig 1:**
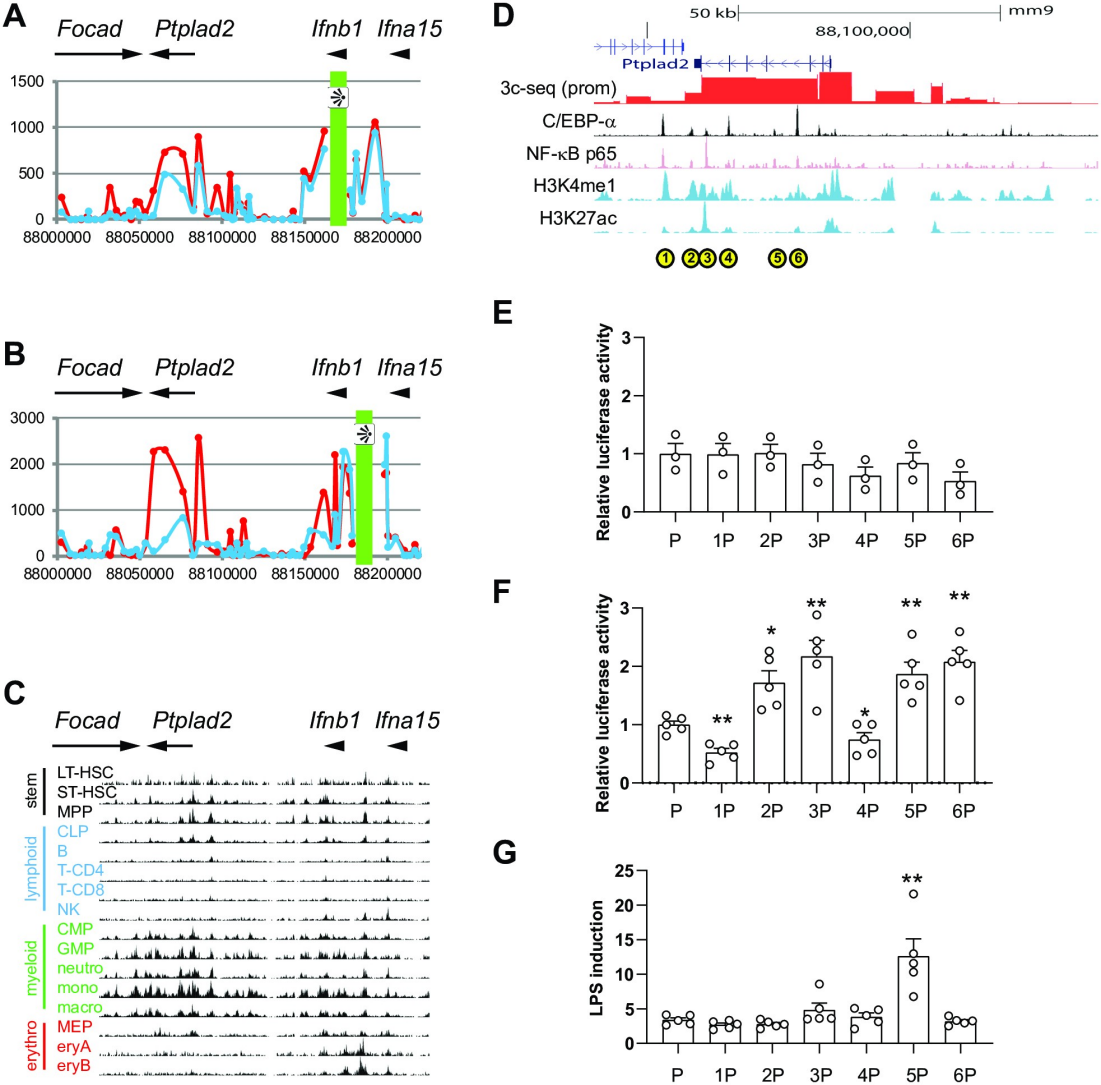
The murine *Ifnb1* gene loops to a myeloid super-enhancer in *Ptplad2*. **(*A* and *B*)** 3c-seq analysis of the *Ptplad2*/*Ifnb1* locus using (*A*) *Ifnb1* or (*B*) the ICE regulatory region as viewpoints in activated WT (blue line) or *Trim33*^-/-^ (red line) macrophages. Genes are indicated above the graph, and position on chromosome 4 (mouse genome version mm9) is indicated below the graph. **(*C*)** Normalized signal from H3K4me1 ChIP-seq experiments performed in hematopoietic sub-populations (B: B lymphocytes, CLP: common lymphoid progenitors, CMP: common myeloid progenitors, eryA: erythroblasts A, eryB: erythroblasts B, GMP: granulocyte monocyte progenitors, LT-HSC: long term hematopoietic stem cells, macro: macrophages, MEP: megakaryocyte erythrocyte progenitors, mono: monocytes, MPP: multipotent progenitors, neutron: neutrophils, NK: natural killer cells, ST-HSC: short term hematopoietic stem cells, T-CD4: CD4+ T lymphocytes, T-CD8: CD8+ T lymphocytes). Data from [40]. **(*D*)** Signal from ChIP-seq experiments targeting different transcription factors and histone marks over the *Ptplad2* gene. The results of the 3C-seq experiment from the *Ifnb1* promoter viewpoint is shown in red. Identified enhancers are indicated with yellow circles, and numbered. Data from [41] (C/EBPα), [42] (NF-κB p65), and [43] (H3K4me1 and H3K27ac). **(*E*)** Plasmids encoding firefly luciferase under the control of the *Ifnb1* promoter (P) flanked or not with the enhancers identified above, each labelled with its number, were transfected into NIH3T3 cells together with a plasmid coding for Renilla luciferase under the CMV promoter. After 30 hrs, luciferase levels were measured. Results are expressed as the ratio of firefly to Renilla luciferase, normalized to the mean value of P. **(*F*)** Plasmids encoding firefly luciferase under the control of the *Ifnb1* promoter (P) flanked or not with the enhancers identified above, each labelled with its number, were transfected into RAW264.7 cells together with a plasmid coding for NanoLuc luciferase under the thymidine kinase promoter. After 30 hrs, luciferase levels were measured. Results are expressed as the ratio of firefly to NanoLuc luciferase, normalized to the mean value of P. **(*G*)** Same experiment as in *F*, except that RAW264.7 cells were treated or not with 100 ng/ml LPS for the last 8 hrs. Results are expressed as the ratio of relative luciferase activity between LPS treated versus non-treated cells. For **(*E*), (*F*)**, and **(*G*)**, results are presented as mean ± s.e.m with open circles showing individual values, each in triplicate (n = 3 to 5). *: p<0.05; **: p<0.01 (two-sided ratio paired t test).

Using chromosome conformation capture experiments followed by deep sequencing (3C-seq, [38]), we have shown that the DNA loop between ICE and the *Ifnb1* gene was not modified in *Trim33*^-/-^ macrophages [24]. To identify distant regions that could participate in the transcriptional regulation of *Ifnb1* in macrophages, we analyzed these data on a larger scale (all accession numbers of publicly available datasets used in this study are provided in S1 Table). We detected an interaction between the *Ifnb1* promoter and a region located around 100 kb downstream, overlapping the *Ptplad2* gene (also called *Hacd4*, Fig 1*A*). This interaction was stronger in *Trim33*^-/-^ than in WT activated macrophages, which correlated with an increased transcription of *Ifnb1*. Interestingly, this 100 kb downstream region also interacted with ICE, an interaction that was also stronger in *Trim33*^-/-^ than in WT activated macrophages (Fig 1*B*). Because the looped region is large (around 30 kb), we explored whether it was predicted as a super-enhancer [39]. Two studies established the genomic distribution of super-enhancers in macrophages from ChIP-seq data targeting either C/EBPα [39] or a combination of PU.1, C/EBPα, JunB, and NF-κB p65 (RelA) [40]. Both predicted a super-enhancer overlapping *Ptplad2*, with a size of 33 kb for the former, and 103 kb for the latter (S1D Fig). This super-enhancer might be myeloid-specific, as it was not predicted in embryonic stem cells (Oct4/Sox2/Nanog ChIP-seq), myotubes (MyoD ChIP-seq), nor T cells (T-bet ChIP-seq) (S1D Fig; data from [39]). Indeed, monomethylation of the lysine 4 of histone H3 (H3K4me1), an epigenetic modification associated with enhancer activity, was detected in this region in hematopoietic stem cells, and then reinforced in the myeloid pathway and erased in the lymphoid and erythroid pathways (Fig 1*C*; data from [41]). This epigenetic profile is consistent with the reinforcement of a large enhancer over *Ptplad2* in myeloid cells.

To identify the potential enhancers present in this super-enhancer, we analyzed data from C/EBPα [42] and NF-κB p65 [43] ChIP-seq, and 2 typical enhancer marks (H3K4me1 and H3K27ac) [44] in murine macrophages. We identified 6 peaks (E1 to E6) in and around the looped region (Fig 1*D*), which bound C/EBPα and NF-κB p65 and showed enrichment in the 2 histone marks. We amplified genomic fragments of around 500 bp centered on each peak and cloned them individually in front of the *Ifnb1* promoter driving transcription of the firefly luciferase in pGL3-basic [24]. Upon transfection into the murine non-myeloid cell line NIH-3T3, none of the fragments increased firefly luciferase expression (Fig 1*E*). Only fragment E1 had an enhancer activity in the murine T lymphoid cell line EL4 (S1E Fig). However, upon transfection into the murine macrophage cell line RAW 264.7, 4 out of 6 fragments (E2, E3, E5, and E6, but not E1) significantly increased luciferase expression around 2 fold when compared to the *Ifnb1* promoter alone (Fig 1*F*). In response to LPS, the *Ifnb1* promoter on its own led to a 3.4 ± 0.3 fold increase in luciferase activity (mean ± s.e.m., Fig 1*G*). Only fragment 5 was able to significantly improve this induction to 12.6 ± 2.5 fold (p < 5.10^−2^) (Fig 1*G*). Because enhancers are orientation-independent, we performed the same experiment with fragments cloned in the reverse orientation. We obtained similar results both for their constitutive (S2A Fig) or LPS-induced (S2B Fig) activities, except for fragment 2, which we therefore did not identify as a *bona fide* enhancer. Altogether, these data show that the increased expression of *Ifnb1* observed in *Trim33*^-/-^ macrophages is associated with, although not fully explained by, an increased looping of *Ifnb1* with a myeloid super-enhancer consisting of 3 individual constitutive enhancers, among which one responds to LPS. We have called this LPS-inducible enhancer FIRE for Far Interferon Regulatory Enhancer.

### A genetic variant in human FIRE is associated with a decreased interferon response in myeloid cells

**Fig 2:**
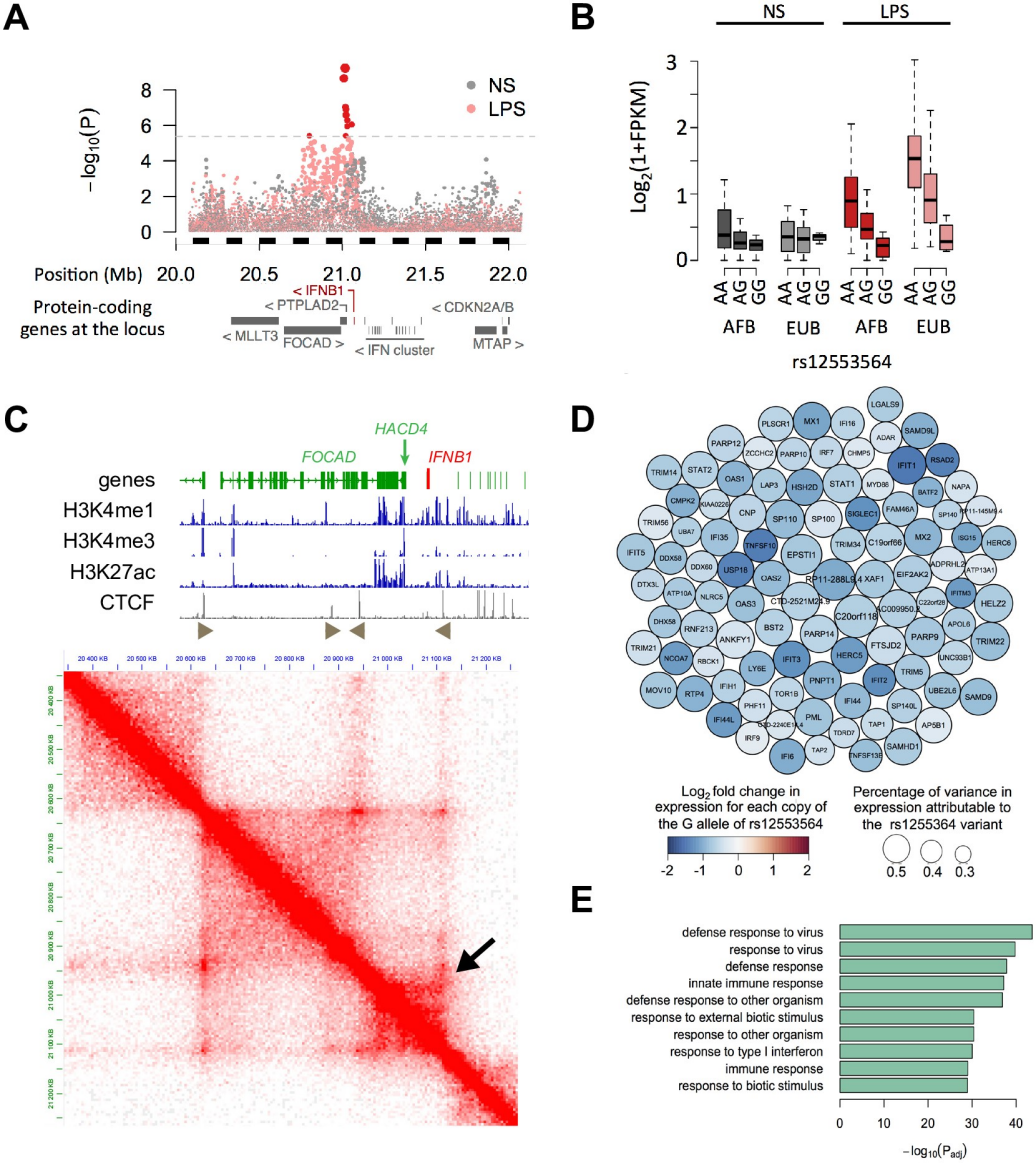
A genetic variant in human FIRE is associated with a decreased interferon response in myeloid cells. **(*A*)** Association of SNPs within 1Mb of *IFNB1* with *IFNB1* expression in non-stimulated (grey) and LPS-stimulated (pink) monocytes. Dotted line indicates the 1% Family wise error rate obtained by permutation. Significant SNPs are highlighted in red. **(*B*)** *IFNB1* expression for each genotype of rs12553564 in 2 populations (AFB: African ancestry from Belgium, EUB: European ancestry from Belgium), in non-stimulated (grey) and LPS-stimulated (red) monocytes. **(*C*)** Hi-C analysis of the FIRE region in THP-1 cells. Genes are indicated on top, with *IFNB1* in red. Histone marks of promoters (H3K4me3) and enhancers (H3K4me1 and H3K27ac) are aligned, as well as oriented CTCF peaks. The black arrow indicates the loop containing *IFNB1* and rs12553564. **(*D*)** Top 100 genes most strongly associated to rs12553564 upon LPS stimulation. Each gene is represented by a circle colored according to the fold change in expression between both alleles of the variant. Size reflects the percentage of variance in gene expression accounted for by the variant. **(*E*)** Functional enrichments of IFNB1 trans–regulated genes.–Log_10_(adjusted p-values) are reported for the top 10 most enriched GO categories.

We and others have identified single nucleotide polymorphisms (SNPs) in the human population that are associated with differential expression of *IFNB1* in LPS-activated monocytes [45,46]. These SNPs are therefore called expression quantitative trait loci (eQTLs). Because such eQTLs might give indications on DNA regions that regulate expression of *IFNB1*, we precisely mapped them in a region covering 2 Mb around *IFNB1*. We performed this analysis jointly on individuals of African or European descent, in order to break population-specific haplotypic blocks, and allow a finer resolution of the mapping of causal variants [47]. The peak of the association was located over the *PTPLAD2* gene, including a set of *17* SNPs significantly correlated with the expression level of *IFNB1* in LPS activated monocytes (Fig 2*A*, p < 4.2 x 10^−6^, corresponding to a family wise error rate of 1%). The strongest association with *IFNB1* expression was observed for variants rs12553564 and rs12551341(p<5.8 x 10^−10^, R^2^ = 19%, Fig 2*B*), which are in perfect linkage disequilibrium (LD). No further association was found between genetic variants and *IFNB1* expression when conditioning on rs12553564 (S3A Fig), suggesting that variants in low LD with rs12553564 do not contribute to the variability in *IFNB1* expression. Variant rs12553564 is part of a 15 kb haplotypic block containing 15 SNPs in high LD (r^2^ > 0.5), including the previously reported rs2275888 (r^2^ = 0.96) [45]. Among these SNPs, rs12553564 was the only one fulfilling a series of analytical criteria (S2 Table). First, rs12553564 is in a region orthologous to the murine enhancers identified above (namely E5, containing FIRE). Second, rs12553564 is located in a predicted regulatory element as defined by the *Ensembl* v80 database. Third, rs12553564 is overlapped by experimentally defined transcription factor binding sites established by the Encode consortium. Fourth, the nucleotide affected in rs12553564 is located at a position conserved across mammals as determined with the GERP++ tool (GerpRS score > 2). And fifth, rs12553564 overlaps several transcription factor binding sites that are conserved across mammals. Although these results do not exclude that other linked variants contribute to the observed phenotype, they prompted us to prioritize rs12553564 for further analysis.

Variant rs12553564 is an A to G substitution located on chromosome 9 at position 21,017,241 (genome version GRCh38), in the third intron of *PTPLAD2*. We observed that no super-enhancer was predicted in human myeloid cells over *PTPLAD2* [48]. We analyzed Hi-C data from the THP-1 human monocytic cell line [49], and found that the *PTPLAD2* region loops to *IFNB1* (**arrow in** Fig 2*C*). This loop is included in a topological domain lined by 2 head-to-tail binding sites for CTCF (Fig 2*C*), a classical organization of functionally isolated chromatin domains [50]. As previously reported, the rs12553564 variant was also associated in *trans* with a total of 433 genes (FDR < 0.01, |β_eQTL_| > 0.2, Fig 2*D*, S3 Table), among which 94% are down-regulated. Gene ontology analysis revealed that antiviral response genes were strongly enriched among them (Fold Enrichment > 10.2, *p* < 1.2 x 10^−48^, Fig 2*E*, S4 Table). These genes most probably represent Interferon Stimulated Genes. In addition to LPS, the rs12553564 variant was found to be an eQTL for *IFNB1* in monocytes activated by Pam_3_CSK_4_ (targeting the TLR1/TLR2 receptors) and R848 (targeting the TLR7/TLR8 receptors), but not after infection with an influenza virus (S3B Fig). Upon Pam_3_CSK_4_ activation, but not R848 activation, rs12553564 was also a trans-eQTL for Interferon Stimulated Genes (S3C Fig). These results show that human FIRE is polymorphic, and suggest that it can regulate *IFNB1* expression in myeloid cells in defined conditions of activation.

### Role of C/EBP-β binding in FIRE function

**Fig 3:**
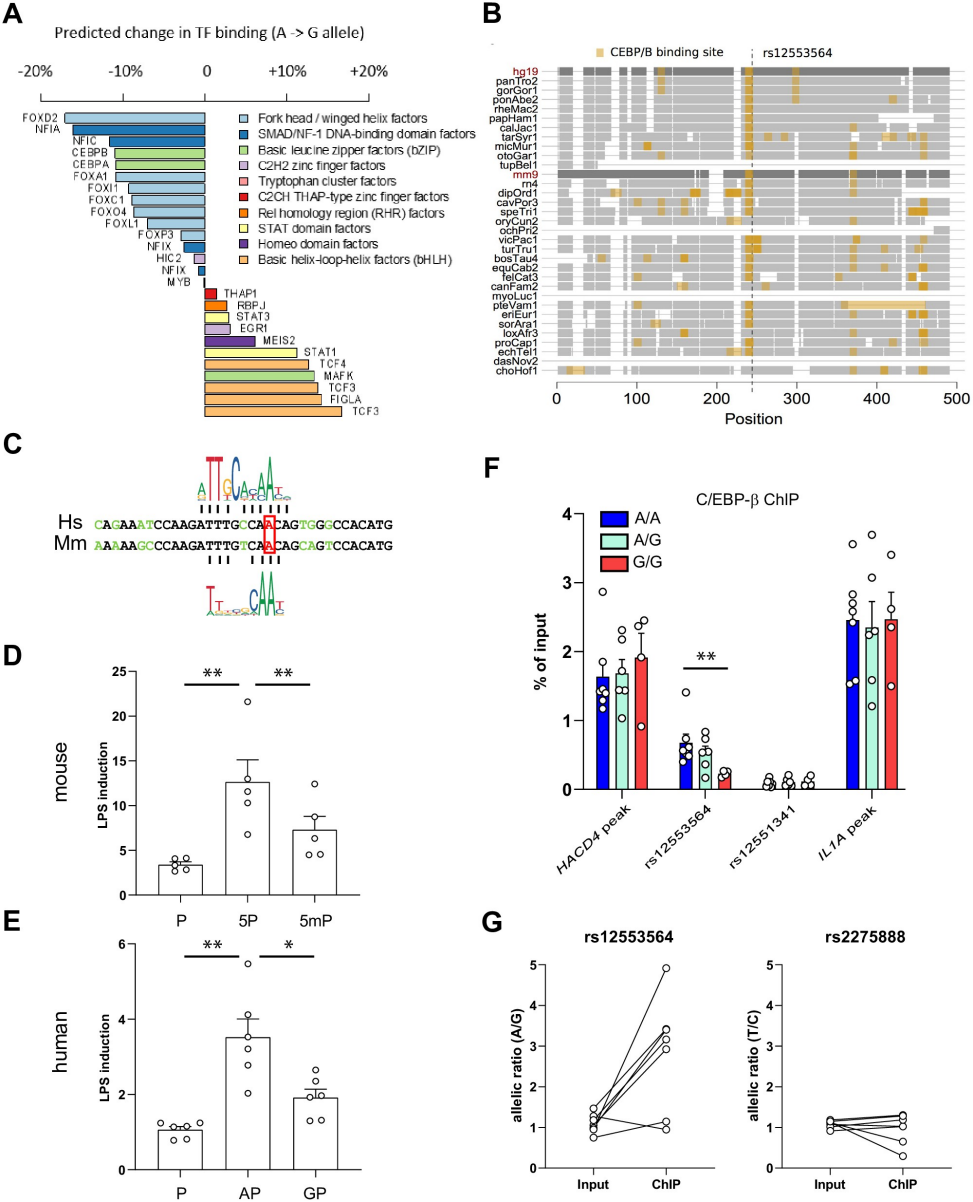
Role of C/EBP-β binding in FIRE function. **(*A*)** Predicted impact of rs12553564 on transcription factor binding. Difference in transcription factor binding scores between the derived (G) and ancestral (A) alleles at the rs12553564 locus. Only transcription factors with a binding score > 85% for either the ancestral or derived allele are reported. Transcription factors are colored according to the tertiary structure of their DNA binding domain. **(*B*)** Cross-species conservation of C/EBP-β binding site at the rs12553564 locus. Sequence alignment of 46 vertebrate species are displayed in a ~500 bp window around the rs12553564 variant. For each genome, only sequences aligning to the human or mice genome are shown, and are displayed by grey boxes. In each genome, matches to the human C/EBP-β motif (>85% of maximal score) are highlighted in orange. **(*C*)** Alignment of the human (Hs) and murine (Mm) sequence around rs12553564 (red box). The Jaspar motifs for human and murine C/EBP-β are indicated above and under the alignment, respectively. **(*D*)** Plasmids encoding firefly luciferase under the control of the *Ifnb1* promoter (P) flanked or not with FIRE (5P) or the A->G mutated FIRE (5mP) were transfected into RAW264.7 cells together with a plasmid coding for NanoLuc luciferase under the thymidine kinase promoter. After 22 hrs, cells were treated or not with 100 ng/ml LPS. After 30 hrs, luciferase levels were measured. Shown are the ratios of relative luciferase activity between LPS treated versus non-treated cells. Results are presented as mean ± s.e.m with open circles showing individual values, each in triplicate (n = 5). **: p<0.01 (two-sided ratio paired t test). **(*E*)** Plasmids encoding firefly luciferase under the control of the human *IFNB1* promoter (P) flanked or not with a 500bp fragment centered on rs12553564 carrying either the A allele (AP) or the G allele (GP) were transfected into RAW264.7 cells together with a plasmid coding for NanoLuc luciferase under the thymidine kinase promoter. After 22 hrs, cells were treated or not with 100 ng/ml LPS. After 30 hrs, luciferase levels were measured. Shown are the ratios of relative luciferase activity between LPS treated versus non-treated cells. Results are presented as mean ± s.e.m with open circles showing individual values, each in triplicate (n=6). *: p<0.05; **: p<0.01 (two-sided ratio paired t test). **(*F*)** C/EBP-β binding as revealed by ChIP on the indicated loci in activated macrophages from healthy donors with the indicated genotypes (A/A: 7 donors, A/G: 6 donors, G/G: 4 donors). *HACD4 peak* and *IL1A peak* indicate non-polymorphic C/EBP-β peaks in *HACD4* (*PTPLAD2*) and *IL1A*, respectively. Results are presented as mean ± s.e.m with open circles showing individual values. **: p<0.01 (two-sided Mann-Whitney test). **(*G*)** Allelic ratio of rs12553564 (left) and rs2275888 (right) before (input) or after (ChIP) C/EBP-β ChIP. ChIP was performed in samples from 7 healthy donors heterozygous for both SNPs. Allelic ratio was determined by allele-specific quantitative Taqman PCR. Connected open circles represent the results for an individual donor.

The association between *IFNB1* expression and a human polymorphism in FIRE suggested that a single nucleotide substitution was sufficient to affect FIRE enhancer function. We sought to determine whether this could be due to decreased binding of a transcription factor. We first assessed the impact of the rs12553564 variant on transcription factor binding motifs. The A to G substitution was predicted to change the binding of 26 transcription factors on the rs12553564 region (Fig 3*A*). Among factors with predicted decreased binding, only 4 were expressed in monocytes (fragments per kb per millions reads (FPKM) > 1), with the highest transcript levels being observed for CEBPB (S4 Fig), the gene coding for the monocyte/macrophage transcription factor C/EBP-β [51]. We analyzed the conservation of C/EBP-β binding motifs in mammals, and found that the rs12553564 C/EBP-β motif was conserved in more than 20 species (Fig 3*B*). The G allele modified an adenine in a high probability position of this motif, as shown by the JASPAR [52] sequence logos for human and murine C/EBP-β (Fig 3*C*).

To study the role of this substitution in the enhancer activity of FIRE, we mimicked rs12553564 in the mouse orthologous sequence by mutating the TTTGTCAAC motif to TTTGTCAGC in the luciferase reporter plasmid. This mutation did not modify the constitutive enhancer activity of FIRE (S5A Fig), but significantly decreased its capacity to be induced by LPS (Fig 3*D*). We also assayed the effect of a 500 bp fragment of human genome centered on rs12553564 and carrying either the A allele or the G allele on the activity of the human *IFNB1* promoter driving expression of firefly luciferase (in pGL4.12). Again, the G allele did not modify the constitutive activity of the enhancer (S5B Fig), but significantly decreased its capacity to be induced by LPS (Fig 3*E*).

We then assessed the binding of C/EBP-β at the rs12553564 position by performing ChIP experiments in myeloid cells from genotyped healthy donors. In accordance with published results [53], the signal obtained in monocytes was too low to reveal a genotype-dependent binding of C/EBP-β on the rs12553564 locus. However, in activated monocyte-derived macrophages, the binding of C/EBP-β was 3 times higher on the A/A allele of rs12553564 than on the G/G allele (0.67 ± 0.13% of input *vs* 0.23 ± 0.02, mean ± s.e.m., 7 donors with the A/A genotype vs 4 with G/G genotype, p < 0.01; Fig 3*F*). The binding on the G/G allele was actually not different from a negative control, which was also the case for rs12551341, regardless of the genotype (Fig 3*F*). Finally, the rs12553564 genotype did not influence C/EBP-β binding on nearby or distant non-mutated C/EBP-β binding loci (Fig 3*F*), demonstrating the specificity of the differential binding on the rs12553564 locus. In order to confirm this differential binding, we performed C/EBP-β ChIP in samples from heterozygous donors, genotyped the resulting DNA with an allele-specific rs12553564 quantitative Taqman PCR, and calculated the A/G allelic ratio in the input and after immunoprecipitation (Fig 3*G*). We found an allelic ratio of 1.1 ± 0.9 in the input, which increased to 2.85 ± 0.52 after ChIP (n = 7, mean ± s.e.m., p < 0.05). All these donors were also heterozygous for rs2275888 (T/C allelic ratio of 1.08 ± 0.03 in the input), but immunoprecipitating C/EBP-β did not enrich one allele of rs2275888 against the other (T/C allelic ratio of 0.97 ± 0.14 after ChIP, p = 0.36) (Fig 3*G*). Altogether, these results show that the rs12553564 polymorphism of FIRE prevents binding of the C/EBP-β transcription factor, and inhibits its LPS inducible enhancer activity.

### Evolution of FIRE in humans

**Fig 4:**
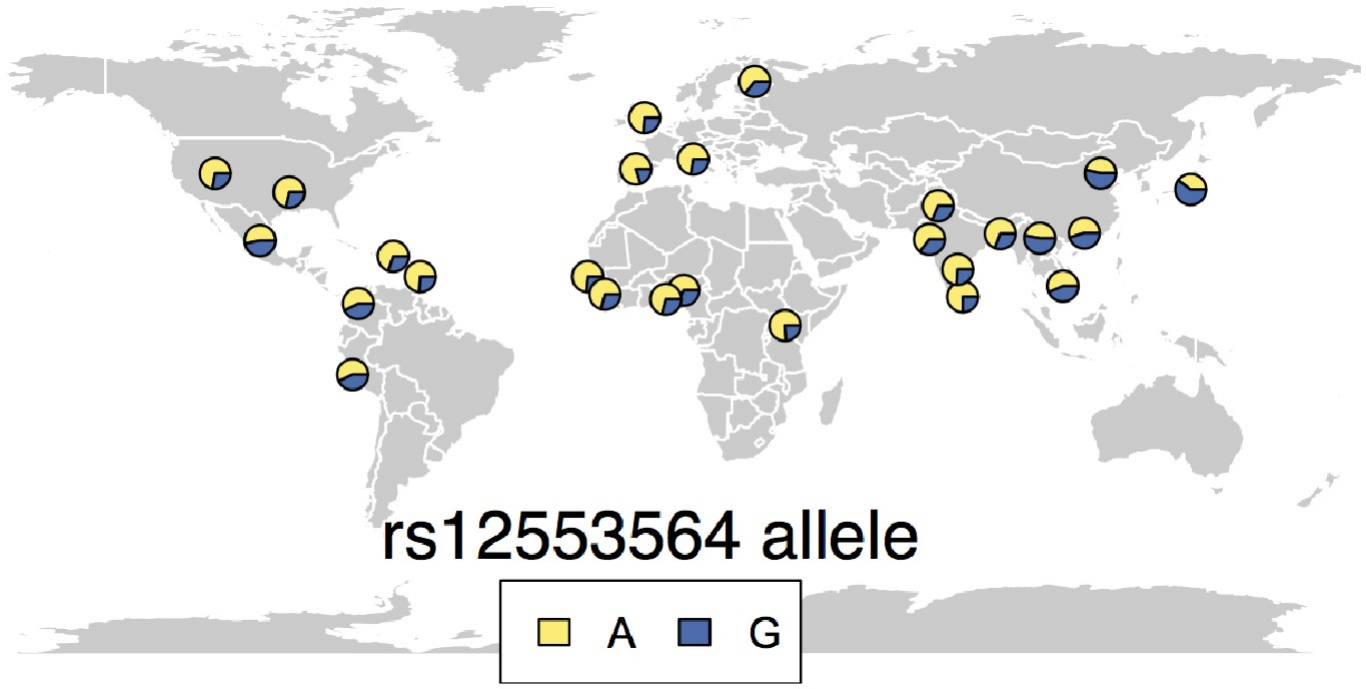
Worldwide allelic frequency at the rs12553564 locus. Geographic repartition of the rs12553564-G allele frequency across populations from 1000 genomes phase 3. The frequency of the G allele (blue) is presented in the different populations as the proportion out of 1 of the depicted pie.

To characterize the evolutionary mode of the FIRE locus, we first considered the geographic distribution of the rs12553564-G allele, based on 1000 genomes data. The rs12553564-G allele was found at high frequency in all populations, suggesting that it appeared before the out-of Africa event ~60 ky ago (Fig 4). The low levels of allelic differentiation at the locus (Global F_ST_ = 0.06, p_emp_ = 0.14), together with the relatively similar haplotype length of both rs12553564 alleles in all populations (|iHS| < 1.52, p_emp_ > 0.20, S5 Table), suggested a lack of positive selection at the locus. We then tested whether balancing selection could explain the high frequency of the rs12553564-G allele. While Tajima’s D at the locus was relatively high in European populations, reaching 1.40 in Toscans (TSI, p_emp_ = 0.04, S5 Table), scan for balancing selection using the β statistics [54] did not highlight any significant deviation from genome wide expectations. To further exclude balancing selection, we extracted 5 kb haplotypes around rs12553564 and used coalescent simulations to date the most recent common ancestor of the haplotypes that segregate at the FIRE locus. We estimated the time to most recent common ancestor at ~623 kY ago (range 451–1010 kY), consistent with genome wide expectations (95% of loci with matched frequency dated between 465 kY and 2 MY), further rejecting the hypothesis of long term balancing selection at the locus. Altogether, our results support that after its appearance >600 kY ago, rs12553564 has evolved neutrally in human populations, suggesting a relaxation of selection in the human lineage.

## Discussion

Through the molecular analysis of a murine genetic model of IFN-β deregulation in myeloid cells, we have identified a myeloid super-enhancer whose looping to the *Ifnb1* gene correlates with increased *Ifnb1* transcription. This super-enhancer contains one LPS inducible enhancer, whose human ortholog carries an *IFNB1* eQTL, i.e. a genetic polymorphism associated with differential *IFNB1* expression. The minor allele disrupts a conserved C/EBP-β binding motif, prevents C/EBP-β binding, and results in decreased *IFNB1* expression levels in activated monocytes. Mimicking the mutation in the murine enhancer directly inhibits its LPS inducible activity. Although we cannot exclude that other human polymorphisms in linkage disequilibrium with rs12553564 could also contribute to the differential expression of *IFNB1*, our results identify a new myeloid-specific *IFNB1* enhancer whose polymorphism controls *IFNB1* expression through binding of C/EBP-β. We have named this enhancer FIRE, for Far *I**FNB1* Regulating Enhancer.

The transcriptional control of *IFNB1* expression has been the subject of intense research, that not only allowed to understand how this physiologically important gene is controlled, but also unveiled fundamental mechanisms of transcriptional regulation. FIRE is a new regulatory region of *IFNB1* expression, with the unique property of being tissue-type specific. The fact that the activity of FIRE depends on the binding of C/EBP-β provides a molecular explanation for tissue specificity. However, we cannot exclude that other regulatory steps, such as DNA loop formation, might also participate in this phenomenon. In any case, FIRE emerges as a molecular target to modify *IFNB1* expression in a tissue-specific manner. This might be of interest, especially because systemic treatments with type I IFN have proved to be highly toxic [55]. Whereas FIRE is embedded in a super-enhancer in mice, this is not the case in humans. Therefore, our data suggest that super-enhancer prediction might not be conserved between species, despite conservation of a major enhancer in the chromatin region. Whether this predictive difference will translate into functional consequences will require further experiments that could shed light on the functional importance of predicted super-enhancers.

The analysis of different monocyte activation pathways revealed a specific pattern of association with rs12553564. The variant was a cis-eQTL for *IFNB1* after activation of the TLR1/TLR2, TLR4, and TLR7/TLR8 pathways, but not after infection with an influenza virus. Virus infection is known to trigger several activation pathways, including the RIG-I/MDA5 pathway, among which some might induce *IFNB1* expression independently from FIRE, or at levels sufficient to mask the effects of rs12553564. In addition, the trans-eQTL effect of rs12553564 was detected after TLR1/TLR2 and TLR4 activation, but not after TLR7/TLR8 activation. It is possible that activation of the latter pathway induces the expression of *IFNA* genes in amounts sufficient to mask the observed difference in *IFNB1* induction. Alternatively, the level of IFN-β induced in R848 activated monocytes from donors carrying the minor allele might be sufficient to achieve maximum target induction. Therefore, although our results show that rs12553564 is associated with *IFNB1* induction in activated monocytes, further experiments are needed to characterize in details its influence in the many IFN-I activation pathways.

Genome wide association studies have provided a wealth of information on human polymorphisms associated with higher order, often disease-linked, phenotypes. However, going from these associations to the involved molecular mechanisms is a complex task. One approach to resolve this issue consists in associating genetic polymorphisms with intermediate processes, such as cellular responses, that drive organismal phenotypes. In this regard, the inducible expression of IFN-β by myeloid cells is both a central process in the viral response, at least for certain viruses, and a cellular process that can be experimentally investigated. Indeed, we were able to show that the binding of C/EBP-β to a distant enhancer could explain the effect of a genetic variant on *IFNB1* expression. However, it is surprising that the FIRE polymorphism is not associated with a human phenotype. We note that this result is in line with the relaxation of selective pressure inferred from the historical analysis of the haplotype. Two hypotheses might explain this observation. First, it is possible that IFN-β expression is robust enough to cope with the expression variation that we observe. Not exclusively, it is possible that quantitative variations of IFN-β produced by activated myeloid cells lead to phenotypic differences that are not deleterious enough to be included in a genome wide association study. It is nonetheless possible that recent pathophysiological situations involving IFN-β, such as radiotherapy, could reveal a functional importance of this human polymorphism.

## Materials and methods

### Ethics statement

Mice experiments were performed in the IRCM animal facility (agreement #B9203202) in compliance with the European Union legislation and the Ethics Committee of the CEA (CETEA agreement #A14_082). For experiments with human monocytes, buffy coats from anonymous healthy donors were obtained at the Etablissement Français du Sang (EFS) after informed consent. Compliance with relevant ethical regulation was controlled by EFS.

### eQTL mapping

Mapping of eQTLs was performed using previously published gene expression and genotype data [46]. Briefly, gene expression data from CD14^+^ monocytes was obtained from 200 individuals of either African (N = 100) or European (N = 100) ancestry, after 6h of stimulation by LPS (N = 184 samples) or rest. After correction for batch effects and technical covariates (GC content and 5’/3’ bias), log_2_-transformed FPKM were used for eQTL mapping. Genotypes were obtained for 9,166 common variants (Minor Allele frequency > 5% in either population) from the *IFNB1* locus (<1Mb from *IFNB1*), based on genotyping with Illumina HumanOmni5-Quad beadchips, exome sequencing using Nextera Rapid Capture Expanded Exome Kits, and imputation with IMPUTE v.2 [56]. Only variants that passed stringent quality criteria were kept for analysis. Details on SNP filtering and gene expression pre-processing can be found in [46]. To map cis-eQTLs of *IFNB1*, we ran the MatrixEQTL R package [57] on both basal and stimulated gene expression, applying an inverse normal rank transformation for each condition and adjusting for the population of origin. Family wise error rate was estimated based on 1000 permutations, retrieving for each permutation the lowest p-value across all SNPs and conditions and choosing a threshold such that a significant p-value is detected in less than 1% of permutations. To assess the trans-effect of the rs12553564 variant, we focused on the LPS stimulated condition and tested the SNP for association will all 12,578 expressed genes, using a linear model with population as a covariate. P-values were corrected using Benjamini Hochberg correction, and a 1% FDR threshold was applied. We further required a minimal effect size (|β|) of 0.2 to consider associations as significant. GO enrichment analyses were performed with GOseq, using the set of all expressed genes as background [58].

### Variant prioritization for follow up

Peak eQTL was defined as the most significant SNP across 5 conditions, when combining both European and African indivudals. r^2^ of nearby genetic variants with rs12553564 was computed across all individuals (100 of African-descent and 100 of Europeans descent). To annotate genetic variants, we retrieved regulatory elements predictions from the Ensembl Regulatory Build v80 [59], and overlapped them with regulatory variants using the Genomic Ranges R package. Similarly, we retrieved a list of Transcription factor binding sites (TFBS) identified by chip-Seq in the Encode Consortium (clustered TFBS peaks v3), and overlapped candidate snps with TFBS position. Conservation across mammals was assessed using base-wise GerpRS [60], and sites with GerpRS>2 were deemed conserved, whereas a GerpRS<2 indicates neutral evolution. GerpRS base-wise mammalian conservation scores were downloaded from the Sidow lab as a measure of local sequence conservation (http://mendel.stanford.edu/sidowlab/downloads/gerp/hg19.GERP_scores.tar.gz).

### Identification of conserved TFBS motifs

All available position frequency matrices for human transcription factors (TF) were retrieved from the JASPAR CORE 2018 database [52], and only the matrix with the highest information content was kept for each TF. Matrices were then converted to position weight matrices using the log probability ratio method with a pseudocount of 0.8 (default value), and assuming an equal prior probability for each possible nucleotide. Multiz 46 way alignments where downloaded from UCSC (http://hgdownload.cse.ucsc.edu/goldenPath/hg19/multiz46way) and used to establish the regions that are orthologous to the *IFNB1* locus (hg19 –chr9:20-22Mb) in mice (mm9) and 44 other verterbrate species. To detect TFBS that are conserved across species around each SNP, we used the searchSeq function from the TFBSTools package to identify TFs with a predicted binding score of >85% of their maximum PWM matrix value across all 46 orthologous sequences in a 21bp window around the SNP, and specifically retained the TFBS that overlapped the human variant. TFBS that were detected in >20 species were considered as conserved. We then assessed the impact of rs12553564 on the predicted binding of the 15 TFs with a conserved binding site at the locus, by comparing the predicted binding score of the two alleles and focusing on the 6 TFs whose binding score decreased by >10%. Of these, only CEBPB was highly expressed (FPKM>100) in our monocytes gene expression data, leading its prioritization for further experiments.

### Mice

Mice with a *Trim33* deletion in myeloid cells were maintained as *Lyz2*^tm1(cre)Ifo/tm1(cre)Ifo^, *Trim33*^fl/fl^, and *Lyz2*^tm1(cre)Ifo/tm1(cre)Ifo^ were used as controls.

### Cell culture

All incubations were performed at 37°C in 5% CO_2_ in a humidified atmosphere. Bone Marrow Derived Macrophages (BMDM) were obtained as described [61]. Briefly, bone marrow cells flushed from the hind limbs of mice were filtered through a 70 μm cell strainer and incubated for 3 h on cell culture treated Petri dishes (1x10 cm dish per animal) in BMDM medium (Iscove’s Modified Dulbecco’s Medium (IMDM, ThermoFisher) supplemented with 10% fetal calf serum (FCS, Sigma), 1% Penicillin/Streptomycin (PS, ThermoFisher), and 10 μM thioglycerol (Sigma)). Non-adherent cells were seeded at 3.5x10^6^ cells per dish (10 cm cell culture treated) in BMDM medium supplemented with 25 ng/ml mouse CSF1 (Miltenyi), and incubated for 7 days with complete medium changes at days 3 and 6. Incubation with LPS (Sigma #L4516) were performed for 24 h at 100 ng/ml in BMDM medium supplemented with 2.5% FCS. RAW 264.7, NIH3T3, and EL4 cells were grown in Dulbecco’s Modified Eagle’s Medium (DMEM, ThermoFisher) supplemented with 10% FCS and 1% PS. In these cell lines, mycoplasma contamination was checked every 6 months with the MycoAlert detection kit (Lonza). Peripheral blood mononuclear cells were prepared by Ficoll density centrifugation (Lymphocytes separation medium, Eurobio) of buffy coats from anonymous healthy donors obtained at the Etablissement Français du Sang (EFS), and frozen in SVF supplemented with 10% dimethyl sulfoxide. Approximately 10^6^ cells were retained for genomic DNA purification (PureLink genomic DNA minikit, ThermoFisher), and genotyped for rs12553564 and rs12551341 with snp genotyping taqman assays (ThermoFisher). After thawing and washing, CD14^+^ cells were purified by labelling with CD14 microbeads and magnetic isolation on LS columns (Miltenyi), according to manufacturer’s instructions. Purity was checked by CD14-PE (Miltenyi) labelling and analysis on a Guava easyCyte 8HT cytometer (Millipore). They were then directly processed for ChIP, or differentiated into macrophages in the presence of 50 ng/ml human M-CSF (Miltenyi) as described [62].

### ChIP

ChIP experiments were performed as described [63]. Briefly, cells were fixed with 1% formaldehyde for 10 min at room temperature, and chromatin was sonicated to 100–500 bp fragments in 1 mM EDTA, 0.5 mM EGTA, 10 mM Tris pH8 with a Bioruptor Pico sonication device (Diagenode). Lysates were precleared with Dynabeads (ThermoFisher), and 1% was sampled as the input. They were then incubated overnight at 4°C with antibodies against mouse CTCF (Millipore #07–729), mouse RAD21 (Abcam ab992), or human C/EBP-β (Abcam ab32358) and then 3 h with saturated Dynabeads. After extensive washing, beads were eluted in 1% sodium dodecyl sulfate, 100 mM NaHCO3, and decrosslinked overnight at 65°C. The immunoprecipitated DNA was purified and used directly in PCR with primers shown in S6 Table, or genotyped with allele-specific quantitative Taqman PCR assays (ThermoFisher, rs12553564 assay ID C____252065_10, rs2275888 assay ID C__16087171_10), or processed for next generation sequencing. In the latter case, they were quantified using Qbit fluorometer (Thermofisher). Sequencing libraries were prepared from 1 ng DNA using the MicroPlex kit (Diagenode) according to the manufacturer’s protocol. DNA was repaired and end-blunted by enzymatic treatment. Stem-loop adaptors with blocked 5’ ends were ligated to the 5’ end of the genomic DNA, leaving a nick at the 3’ end. The 3’ ends of the genomic DNA were extended to complete library synthesis and Illumina-compatible indexes were added through amplification. Libraries were purified using AMPure XP beads (Beckman Coulter) and quantified using Qbit fluorometer. Libraries fragment size distribution was verified using the Bioanalyzer high sensitivity DNA chip (Agilent Technologies). Libraries were mixed in an equimolar pool and a 1% spike-in PhiX Control v3 (Illumina) was added. Clusters were generated and sequenced using a Nextseq 500 instrument (Illumina) in single read mode (75 cycles). Sequences were demultiplexed, quality controlled by the Aozan tool [64], trimmed with Cutadapt 1.5, and aligned on the mm9 version of the mouse genome with Bowtie 2. Peak calling was performed with MACS with default settings, and co-localization of peaks was analyzed with seqMINER [65].

### Luciferase assays

The vector encoding firefly luciferase under the control of the murine *Ifnb1* promoter has been previously described [24], and is based on pGL3-basic. Six DNA fragments of around 500 bp centered on each individual enhancer were obtained by PCR amplification of genomic DNA from WT BMDM with primers designed with Primer3Plus (http://www.bioinformatics.nl/cgi-bin/primer3plus/primer3plus.cgi) (see S6 Table). They were cloned in front the *Ifnb1* promoter, and sequence-verified (Eurofins). The human *IFNB1* promoter was inserted in front of the luciferase gene in pGL4.12 by Sequence and Ligation Independent Cloning (SLIC). A fragment of human genomic DNA centered on rs12553564 was amplified by PCR from THP-1 genomic DNA (allele A) and inserted in front of the promoter by SLIC, and then mutated to the G allele by SLIC. All constructs were sequenced (Eurofins), and primers can be found in S6 Table. RAW264.7, NIH-3T3, and EL4 cells were transfected in triplicate with jetPEI-Macrophage (Polyplus Transfection), Lipofectamine 2000 (ThermoFisher), or Lipofectamine 3000 (ThermoFisher), respectively, according to manufacturers’ instructions. A vector coding for NanoLuc luciferase under the control of the thymidine kinase promoter (or Renilla luciferase under the control of the CMV promoter for NIH3T3 cells) was used to normalize transfection efficiencies. Thirty hours after transfection, both luciferase activities were measured with the Nano-Glo Dual (or Dual-Glo) Luciferase Assay System (Promega), according to manufacturer’s instructions. Where indicated, cells were treated with 100 ng/ml LPS for the last 8 h of incubation. The mean of the values obtained for the promoter alone in n independent experiments was used to normalize individual values. Results are are reported as mean ± s.e.m. of n independent experiments.

### Evolutionary analysis

To evaluate the evolutionary mode of the locus, we focused on populations from the 1000 genomes project phase 2 and performed selection scans on the entirety of chromosome 9, considering global AMOVA-based F_ST_ values, and population-wise iHS, Tajima’s D, and allele frequency correlation (β scores). iHS and Tajima’s D were computed based on 100 kb around the SNP of interest. The derived allele of each SNP was defined based on the 6-EPO alignments and only SNPs with a derived allele frequency (DAF) between 5% and 95% were analysed. iHS scores were normalized in 40 separate bins of DAF. Βeta scores for all 1000G populations were obtained from http://coruscant.itmat.upenn.edu/data/SiewertEA_Full_BetaScores.tar.gz. These β scores are based on a 1 kb window around the SNP (500 bp on each side). For each statistic, an empirical *p*-value measuring evidence for positive/balancing selection at the rs12553564 variant was computed. Namely, the empirical p-value was obtained as the rank of the observed value of F_ST_, Tajima’D, |iHS| or β among all other values observed on chromosome 9. Time of most recent common ancestor (TMRCA) of haplotypes at the rs12553564 locus was estimated using ARGweaver. We downloaded phased VCF from 1000 genomes (1 kG) phase 3 and extracted all variants in a 10 kb region around the rs12553564 locus (5 kb on each side). After filtering out variants with a MAF < 1% across all populations, ARGweaver was run with default values for effective population size of (Neff = 10000), and mutation rate (mu = 2.5e-8), a total of 20 discrete time steps and a maximum time of 200,000 generations. Compression factor of 100 was used to speed up calculations. Genome wide distribution of TMRCA values was obtained by applying ARGweaver with the same parameters to 100 randomly sampled 10 kb regions along the genome.

### Accession numbers

High-throughput sequencing results have been deposited to Gene Expression Omnibus (GEO) with accession number GSE137514. The accession numbers of publicly available data used in this study are detailed in S1 Table.

## Supporting information

S1 Fig **(A)** Alignment of ChIP-seq signals for CTCF, RAD21 and TRIM33 in RAW264.7 cells on the peaks bound by either of these proteins, after clustering with SeqMiner. **(B)** Genome view of ChIP-seq signals for TRIM33, CTCF, and RAD21 around *Ifnb1*. TRIM33/CTCF peaks are boxed in green. **(C)** Enrichment of GO term clusters for genes over-expressed (top) or under-expressed (bottom) in *Ctcf*^-/-^ macrophages treated with LPS for 24 hrs. **(D)** Prediction of super-enhancers in murine myeloid cells (Young lab C/ZEBPa and SE_Hah T0 and T2) as compared to 4 other cell types. **(E)** Plasmids encoding firefly luciferase under the control of the Ifnb1 promoter alone (P) or combined with the 6 murine enhancers were transfected into EL4 cells together with a plasmid coding for NanoLuc luciferase under the control of the thymidine kinase promoter. After 30 hrs, luciferase levels were measured. Results are expressed as the ration of firefly to NanoLuc luciferase, normalized to the mean value of P, and presented as mean +/- s.e.m. with individual experiments shown as open circles (n = 7), each performed in triplicate. *: p<0.05.
(PDF)

S2 Fig **(A)** Plasmids encoding firefly luciferase under the control of the Ifnb1 promoter alone (P) or combined with the 6 murine enhancers in reverse orientation (ExrevP) were transfected into RAW264.7 cells together with a plasmid coding for NanoLuc luciferase under the control of the thymidine kinase promoter. After 30 hrs, luciferase levels were measured. Results are expressed as the ratio of firefly to NanoLuc luciferase, normalized to the mean value of P, and presented as mean +/- s.e.m. with individual experiments shown as open circles (n = 5), each performed in triplicate. **(B)** Same as (A) except that cells were treated with 100 ng/ml LPS for the last 8 hrs. Results are expressed as the ration of the values obtained with LPS to the values without LPS (n = 5). *: p<0.05; **: p<0.01; ***: p<0.001 (ratio paired t test).
(PDF)

S3 Fig **(A)** Association of SNPs within 1Mb of *IFNB1* with *IFNB1* expression in non-stimulated (grey) and LPS-stimulated (pink) monocytes after conditioning on rs12553564. Dotted line indicates the 1% Family wise error rate obtained by permutation. **(B)** Expression of *IFNB1* in monocytes from healthy donors with different rs12553564 genotypes, as indicated, activated or not by different inducers as depicted above the graph. **(C)** Expression of 3 IFNB1 targets (IFIT1, MX1, and STAT1) in monocytes from healthy donors with different rs12553564 genotypes, as indicated, activated or not by different inducers as depicted above the graph.
(PDF)

S4 Fig Gene expression of transcription factors in unstimulated (grey) or LPS stimulated (red) human monocytes. Results were analyzed from RNA-seq data described in Quach et al., 2016 [45].
(PDF)

S5 Fig **(A)** Plasmids encoding firefly luciferase under the control of the murine *Ifnb1* promoter alone (P) or combined with FIRE (5P) or the mutated FIRE mimicking rs12553564 (5mP) were transfected into RAW264.7 cells together with a plasmid coding for NanoLuc luciferase under the control of the tymidine kinase promoter. After 30 hrs, luciferase levels were measured. Results are expressed as the ratio of firefly to NanoLuc luciferase, normalized to P, and presented as mean +/- sem with individual values shown as open circles (n = 5). *: p<0.05; ns: not significant. **(B)** Same experiment with plasmids encoding firefly luciferase under the control of the human *IFNB1* promoter alone (P) or combined with a human genomic fragment centered on rs12553564 and carrying the A allele (AP) or the G allele (GP).
(PDF)

S1 Table Accession numbers and references of publicly available data used in the manuscript. (DOCX)

S2 Table Analysis of genetic variants associated with variation of *IFNB1* expression in LPS-activated monocytes. (XLSX)

S3 Table List of the 433 genes regulated in trans by rs12553564. (XLSX)

S4 Table GO analysis of the 433 genes regulated in trans by rs12553564. (XLSX)

S5 Table Evolutionary analysis of rs12553564 in different populations from 1000 genomes. (XLSX)

S6 Table Primers used in the manuscript. (XLSX)
