# Supplementary material for: A genetic variant controls interferon-β gene expression in human myeloid cells by preventing C/EBP-β binding on a conserved enhancer
Source: PLoS Genet. 2020 Nov 4;16(11):e1009090. doi: 10.1371/journal.pgen.1009090 (PMC7641354; doi:10.1371/journal.pgen.1009090)
Supplement: S1 Fig — (A) Alignment of ChIP-seq signals for CTCF, RAD21 and TRIM33 in RAW264.7 cells on the peaks bound by either of these proteins, after clustering with SeqMiner. (B) Genome view of ChIP-seq signals for TRIM33, CTCF, and RAD21 around Ifnb1. TRIM33/CTCF peaks are boxed in green. (C) Enrichment of GO term clusters for genes over-expressed (top) or under-expressed (bottom) in Ctcf-/- macrophages treated with LPS for 24 hrs. (D) Prediction of super-enhancers in murine myeloid cells (Young lab C/ZEBPa and SE_Hah T0 and T2) as compared to 4 other cell types. (E) Plasmids encoding firefly luciferase under the control of the Ifnb1 promoter alone (P) or combined with the 6 murine enhancers were transfected into EL4 cells together with a plasmid coding for NanoLuc luciferase under the control of the thymidine kinase promoter. After 30 hrs, luciferase levels were measured. Results are expressed as the ration of firefly to NanoLuc luciferase, normalized to the mean value of P, and presented as mean +/- s.e.m. with individual experiments shown as open circles (n = 7), each performed in triplicate. *: p<0.05. (PDF) [file pgen.1009090.s001.pdf]

A

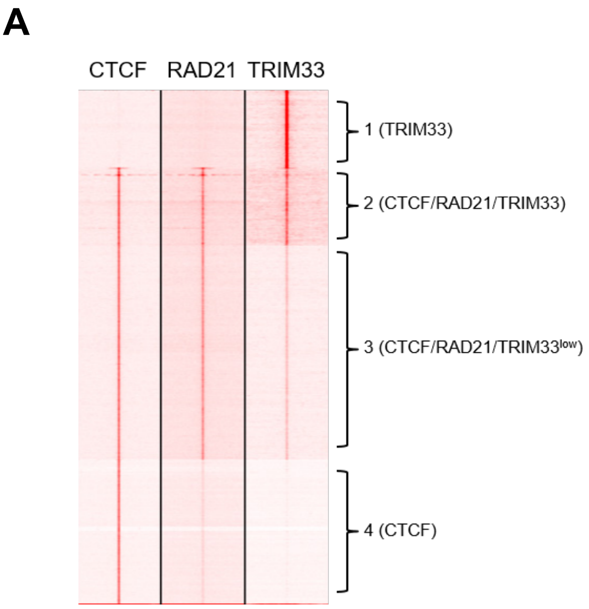

B

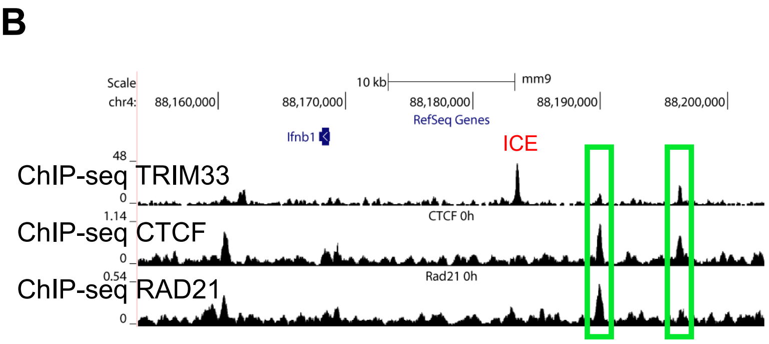

C

Cluster	Enrichment	GO terms	genes	P value
**1**	**2.31**	chemokine-mediated signaling pathway<br>chemotaxis<br>inflammatory response	4<br>5<br>6	$2.2x10^{-3}$<br>$2.3x10^{-3}$<br>$2.3x10^{-2}$

GO term enrichment in genes over-expressed in activated *Ctcf*$^{-/-}$ macrophages

Cluster	Enrichment	GO terms	genes	P value
**1**	**4.36**	cellular response to interferon γ<br>cellular response to interferon β<br>defense response to protozoan<br>adhesion of symbiont to host	7<br>5<br>4<br>3	$5.5x10^{-7}$<br>$3.2x10^{-5}$<br>$3.1x10^{-4}$<br>$6.8x10^{-4}$
**2**	**3.98**	inflammatory response<br>immune response<br>cell chemotaxis<br>chemokine-mediated signaling pathway<br>chemotaxis	12<br>9<br>5<br>4<br>5	$3.3x10^{-7}$<br>$2.7x10^{-5}$<br>$4.0x10^{-4}$<br>$1.9x10^{-3}$<br>$1.9x10^{-3}$
**3**	**2.85**	response to virus<br>innate immune response<br>defense response to virus<br>negative regulation of viral genome replication	6<br>8<br>5<br>3	$3.5x10^{-5}$<br>$2.0x10^{-3}$<br>$6.4x10^{-3}$<br>$8.8x10^{-3}$

GO term enrichment in genes under-expressed in activated *Ctcf*$^{-/-}$ macrophages

D

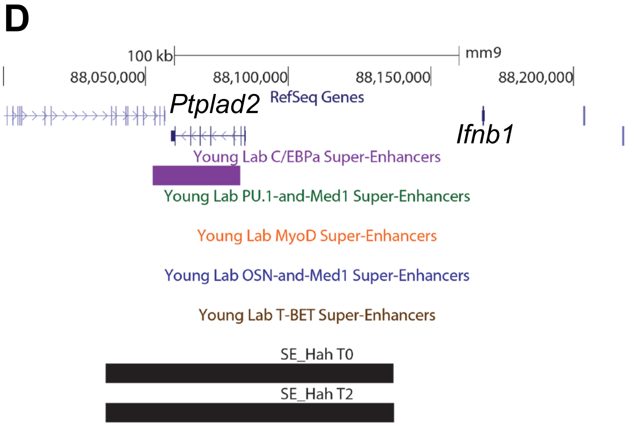

E

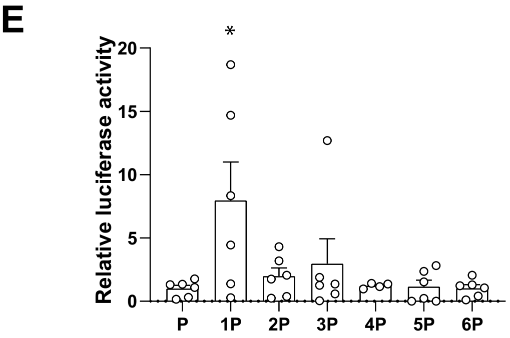

Figure S1:
(A) Alignment of ChIP-seq signals for CTCF, RAD21 and TRIM33 in RAW264.7 cells on the peaks bound by either of these proteins, after clustering with SeqMiner. **(B)** Genome view of ChIP-seq signals for TRIM33, CTCF, and RAD21 around *Ifnb1*. TRIM33/CTCF peaks are boxed in green. **(C)** Enrichment of GO term clusters for genes over-expressed (top) or under-expressed (bottom) in *Ctcf*-/- macrophages treated with LPS for 24 hrs. **(D)** Prediction of super-enhancers in murine myeloid cells (Young lab C/ZEBPa and SE_Hah T0 and T2) as compared to 4 other cell types. **(E)** Plasmids encoding firefly luciferase under the control of the Ifnb1 promoter alone (P) or combined with the 6 murine enhancers were transfected into EL4 cells together with a plasmid coding for NanoLuc luciferase under the control of the thymidine kinase promoter. After 30 hrs, luciferase levels were measured. Results are expressed as the ration of firefly to NanoLuc luciferase, normalized to the mean value of P, and presented as mean +/- s.e.m. with individual experiments shown as open circles (n=7), each performed in triplicate. *: p<0.05Enhancer activity of the 6 DNA fragments in the murine EL4 cell line in a luciferase reporter experiment. *: p<0.05
