# Supplementary material for: A genetic variant controls interferon-β gene expression in human myeloid cells by preventing C/EBP-β binding on a conserved enhancer
Source: PLoS Genet. 2020 Nov 4;16(11):e1009090. doi: 10.1371/journal.pgen.1009090 (PMC7641354; doi:10.1371/journal.pgen.1009090)
Supplement: S2 Fig — (A) Plasmids encoding firefly luciferase under the control of the Ifnb1 promoter alone (P) or combined with the 6 murine enhancers in reverse orientation (ExrevP) were transfected into RAW264.7 cells together with a plasmid coding for NanoLuc luciferase under the control of the thymidine kinase promoter. After 30 hrs, luciferase levels were measured. Results are expressed as the ratio of firefly to NanoLuc luciferase, normalized to the mean value of P, and presented as mean +/- s.e.m. with individual experiments shown as open circles (n = 5), each performed in triplicate. (B) Same as (A) except that cells were treated with 100 ng/ml LPS for the last 8 hrs. Results are expressed as the ration of the values obtained with LPS to the values without LPS (n = 5). *: p<0.05; **: p<0.01; ***: p<0.001 (ratio paired t test). (PDF) [file pgen.1009090.s002.pdf]

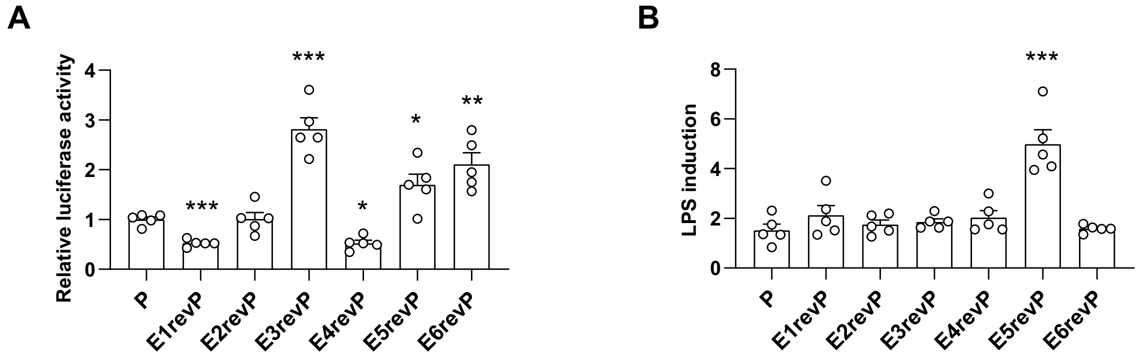

Figure S2: Validation of murine enhancers in reverse orientation
(A) Plasmids encoding firefly luciferase under the control of the Ifnb1 promoter alone (P) or combined with the 6 murine enhancers in reverse orientation (ExrevP) were transfected into RAW264.7 cells together with a plasmid coding for NanoLuc luciferase under the control of the thymidine kinase promoter. After 30 hrs, luciferase levels were measured. Results are expressed as the ration of firefly to NanoLuc luciferase, normalized to the mean value of P, and presented as mean +/- s.e.m. with individual experiments shown as open circles (n=5), each performed in triplicate. **(B)** Same as (A) except that cells were treated with 100 ng/ml LPS for the last 8 hrs. Results are expressed as the ration of the values obtained with LPS to the values without LPS (n=5). *: $p<0.05$; **: $p<0.01$; ***: $p<0.001$ (ratio paired t test).
