# Supplementary material for: A genetic variant controls interferon-β gene expression in human myeloid cells by preventing C/EBP-β binding on a conserved enhancer
Source: PLoS Genet. 2020 Nov 4;16(11):e1009090. doi: 10.1371/journal.pgen.1009090 (PMC7641354; doi:10.1371/journal.pgen.1009090)
Supplement: S3 Fig — (A) Association of SNPs within 1Mb of IFNB1 with IFNB1 expression in non-stimulated (grey) and LPS-stimulated (pink) monocytes after conditioning on rs12553564. Dotted line indicates the 1% Family wise error rate obtained by permutation. (B) Expression of IFNB1 in monocytes from healthy donors with different rs12553564 genotypes, as indicated, activated or not by different inducers as depicted above the graph. (C) Expression of 3 IFNB1 targets (IFIT1, MX1, and STAT1) in monocytes from healthy donors with different rs12553564 genotypes, as indicated, activated or not by different inducers as depicted above the graph. (PDF) [file pgen.1009090.s003.pdf]

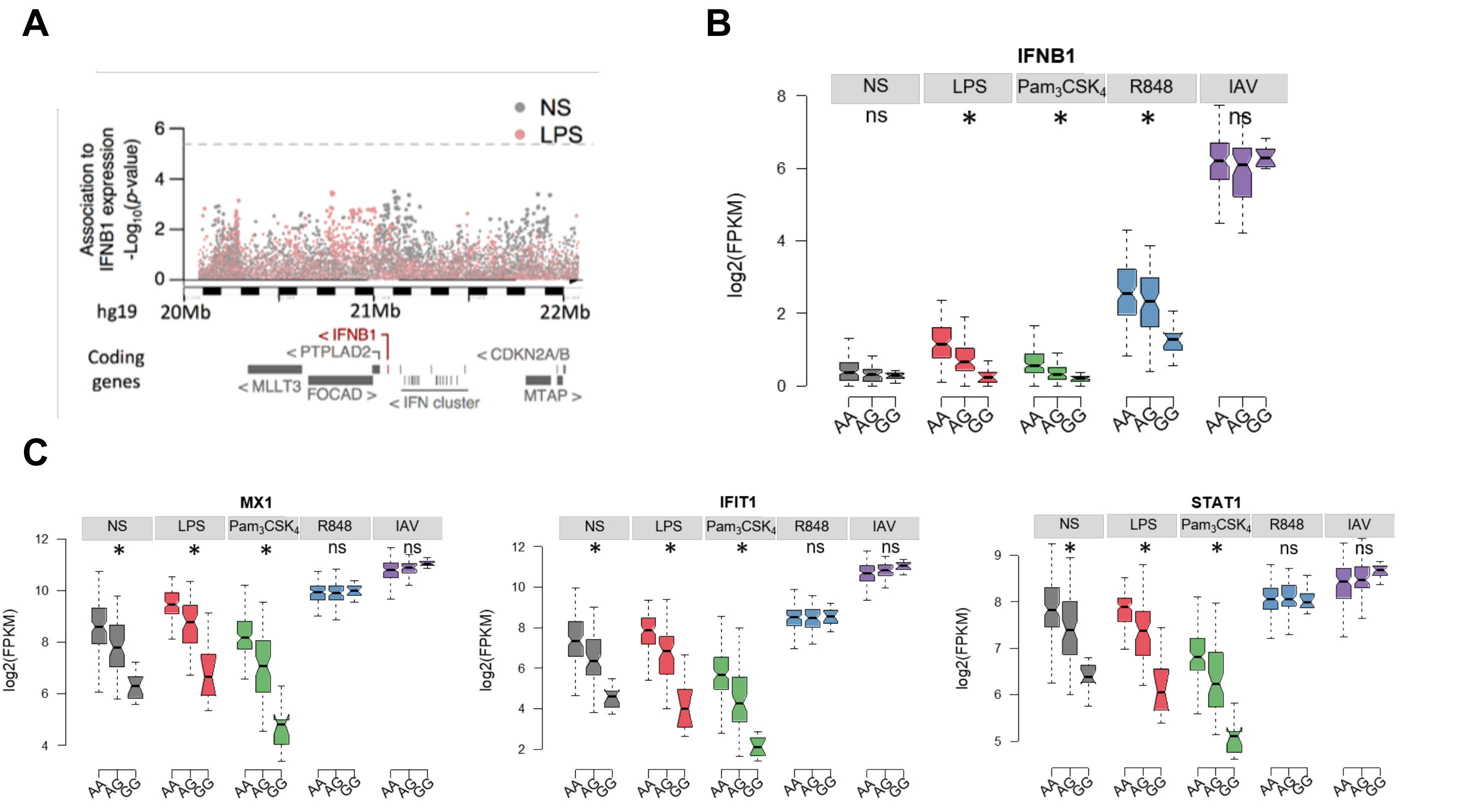

Figure S3

(A) Association of SNPs within 1Mb of IFNB1 with IFNB1 expression in non-stimulated (grey) and LPS-stimulated (pink) monocytes after conditioning on rs12553564. Dotted line indicates the 1% Family wise error rate obtained by permutation. **(B)** Expression of IFNB1 in monocytes from healthy donors with different rs12553564 genotypes, as indicated, activated or not by different inducers as depicted above the graph. **(C)** Expression of 3 IFNB1 targets (IFIT1, MPX1, and STAT1) in monocytes from healthy donors with different rs12553564 genotypes, as indicated, activated or not by different inducers as depicted above the graph.
