# Supplementary material for: A genetic variant controls interferon-β gene expression in human myeloid cells by preventing C/EBP-β binding on a conserved enhancer
Source: PLoS Genet. 2020 Nov 4;16(11):e1009090. doi: 10.1371/journal.pgen.1009090 (PMC7641354; doi:10.1371/journal.pgen.1009090)
Supplement: S4 Fig — Results were analyzed from RNA-seq data described in Quach et al., 2016 [45]. (PDF) [file pgen.1009090.s004.pdf]

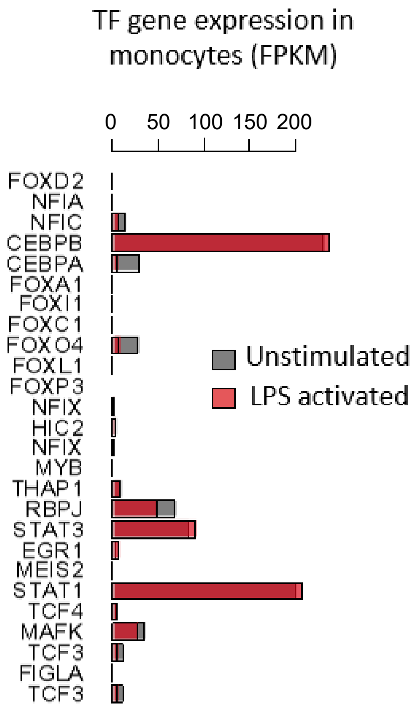

Figure S4:
Gene expression of transcription factors in unstimulated (grey) or LPS stimulated (red) human monocytes. Results were analyzed from RNA-seq data described in Quach et al., 2016 (46).
