# Supplementary material for: A genetic variant controls interferon-β gene expression in human myeloid cells by preventing C/EBP-β binding on a conserved enhancer
Source: PLoS Genet. 2020 Nov 4;16(11):e1009090. doi: 10.1371/journal.pgen.1009090 (PMC7641354; doi:10.1371/journal.pgen.1009090)
Supplement: S5 Fig — (A) Plasmids encoding firefly luciferase under the control of the murine Ifnb1 promoter alone (P) or combined with FIRE (5P) or the mutated FIRE mimicking rs12553564 (5mP) were transfected into RAW264.7 cells together with a plasmid coding for NanoLuc luciferase under the control of the tymidine kinase promoter. After 30 hrs, luciferase levels were measured. Results are expressed as the ratio of firefly to NanoLuc luciferase, normalized to P, and presented as mean +/- sem with individual values shown as open circles (n = 5). *: p<0.05; ns: not significant. (B) Same experiment with plasmids encoding firefly luciferase under the control of the human IFNB1 promoter alone (P) or combined with a human genomic fragment centered on rs12553564 and carrying the A allele (AP) or the G allele (GP). (PDF) [file pgen.1009090.s005.pdf]

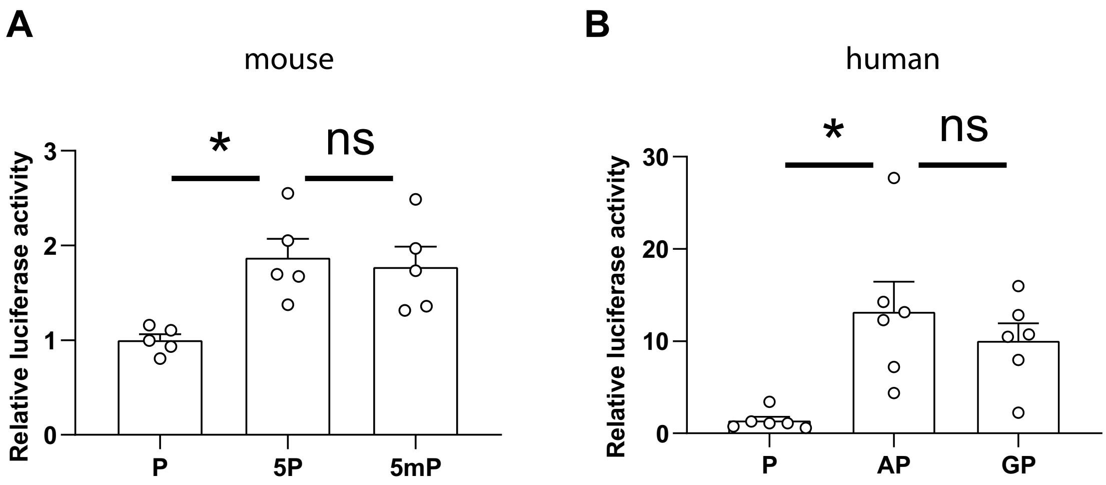

Figure S5:
(A) Plasmids encoding firefly luciferase under the control of the murine *Ifnb1* promoter alone (P) or combined with FIRE (5P) or the mutated FIRE mimicking rs12553564 (5mP) were transfected into RAW264.7 cells together with a plasmid coding for NanoLuc luciferase under the control of the tymidine kinase promoter. After 30 hrs, luciferase levels were measured. Results are expressed as the ratio of firefly to NanoLuc luciferase, normalized to P, and presented as mean +/- sem with individual values shown as open circles (n=5). *: p<0.05; ns: not significant. **(B)** Same experiment with plasmids encoding firefly luciferase under the control of the human *IFNB1* promoter alone (P) or combined with a human genomic fragment centered on rs12553564 and carrying the A allele (AP) or the G allele (GP).
