# Supplementary material for: A genetic variant controls interferon-β gene expression in human myeloid cells by preventing C/EBP-β binding on a conserved enhancer
Source: PLoS Genet. 2020 Nov 4;16(11):e1009090. doi: 10.1371/journal.pgen.1009090 (PMC7641354; doi:10.1371/journal.pgen.1009090)
Supplement: S1 Table — (DOCX) [file pgen.1009090.s006.docx]

**S1 Table : Accession numbers of high-throughput sequencing results used in the paper**

| **Experiment** | **Cell** | **Target or viewpoint** | **Accession number** | **reference** |
| --- | --- | --- | --- | --- |
| 3c-seq | BMDM | ICE and *Ifnb1* gene | GSE73322 | Ferri et al., 2015 |
| ChIP-seq | RAW264.7 | TRIM33 | GSM1067639 | Ferri et al., 2015 |
| ChIP-seq | RAW264.7 | CTCF and RAD21 | GSE137514 | This paper |
| ChIP-seq | Hematopoietic progenitors | H3K4me1 and H3K27ac | GSE60103 | Lara-Astiaso et al., 2014 |
| ChIP-seq | BMDM | C/EBPα | GSE50565 | Zhang et al., 2013 |
| ChIP-seq | BMDM | NF-κB p65 | GSM611116 | Barish et al., 2010 |
| ChIP-seq | BMDM | H3K4me1 and H3K27ac | Encode |  |
| RNA-seq | Monocytes |  | EGAS00001001895 | Quach et al., 2016 |

References

1. Ferri, F., Parcelier, A., Petit, V., Gallouet, A.-S., Lewandowski, D., Dalloz, M., Van Den Heuvel, A., Kolovos, P., Soler, E., Squadrito, M.L., et al. (2015). TRIM33 switches off Ifnb1 gene transcription during the late phase of macrophage activation. Nat. Commun. *6*, 8900.

2. Lara-Astiaso, D., Weiner, A., Lorenzo-Vivas, E., Zaretsky, I., Jaitin, D.A., David, E., Keren-Shaul, H., Mildner, A., Winter, D., Jung, S., et al. (2014). Chromatin state dynamics during blood formation. Science (80-. ). *345*, 943–949.

3. Zhang, H., Alberich-Jorda, M., Amabile, G., Yang, H., Staber, P.B., Di Ruscio, A., Welner, R.S., Ebralidze, A., Zhang, J., Levantini, E., et al. (2013). Sox4 Is a Key Oncogenic Target in C/EBPα Mutant Acute Myeloid Leukemia. Cancer Cell *24*, 575–588.

4. Barish, G.D., Yu, R.T., Karunasiri, M., Ocampo, C.B., Dixon, J., Benner, C., Dent, A.L., Tangirala, R.K., and Evans, R.M. (2010). Bcl-6 and NF-κB cistromes mediate opposing regulation of the innate immune response. Genes Dev. *24*, 2760–2765.

5. Quach, H., Rotival, M., Pothlichet, J., Loh, Y.-H.E., Dannemann, M., Zidane, N., Laval, G., Patin, E., Harmant, C., Lopez, M., et al. (2016). Genetic Adaptation and Neandertal Admixture Shaped the Immune System of Human Populations. Cell *167*, 643–656.
